# Anterior teeth root inclination prediction derived from digital models: A comparative study of plaster study casts and CBCT images 

**DOI:** 10.4317/jced.55180

**Published:** 2018-11-01

**Authors:** Mahmoud Dastoori, Joseph P. Bouserhal, Demetrios J. Halazonetis, Athanasios E. Athanasiou

**Affiliations:** 1 Former resident, Department of Orthodontics, Hamdan Bin Mohammed College of Dental Medicine, Mohammed Bin Rashid University of Medicine and Health Sciences, Dubai, UAE; Orthodontic Specialist, London Dental Clinic, Dubai, UAE; 2 Professor, Department of Orthodontics, Faculty of Dental Medicine, Saint Joseph University of Beirut, Beirut, Lebanon; 3 Professor and Chairman, Department of Orthodontics, School of Dentistry, National and Kapodistrian University of Athens, Athens, Greece; 4 Dean and Professor of Orthodontics, Hamdan Bin Mohammed College of Dental Medicine, Mohammed Bin Rashid University of Medicine and Health Sciences, Dubai, UAE

## Abstract

**Background:**

To assess the accuracy of digital models generated using commercially available software to predict anterior teeth root inclination characteristics and compare the results to relevant data obtained from CBCT images.

**Material and Methods:**

Following sample size calculation and after application of inclusion and exclusion criteria, pre-treatment maxillary and mandibular plaster models and the corresponding CBCT scans of 31 patients attending a private orthodontic clinic were selected. The subjects were 10 males and 21 females with age range 12 to 40 years. Plaster models were scanned using the high resolution mode of an Ortho Insight 3D scanner and CBCT scans were taken using a Kodak 9500 Cone Beam 3D System machine. The teeth on the digital scans were segmented and virtual roots were predicted and constructed by the Ortho Insight 3D software. The long axes of the predicted roots and the actual roots, as segmented from the CBCT images, were computed using best-fit lines. The inter-axis angle was used to assess error in root inclination prediction by the software. Mann-Whitney and Kruskal-Wallis tests were used. Intra-examiner error was evaluated using the Bland-Altman method.

**Results:**

The maximum disparity in angle between images derived from digital models and CBCT data was almost 40 degrees (upper left canine). The upper and lower canines produced the worst results, followed by the lower lateral incisors. The upper central incisors showed the best results, although the maximum angle of difference exceeded 20 degrees (with the median around 8 degrees).

**Conclusions:**

Root morphology imaging prediction is not a primary function of this software and this study confirmed its limitation as a sole tool in routine clinical applications. At present these predictions cannot be considered accurate or reliable unless correlated clinically with a radiographic image.

** Key words:**Digital models, CBCT, tooth root inclination prediction software.

## Introduction

Nowadays 3D digital model scanners are increasingly incorporated in orthodontic offices and benchtop scanners can now construct a digital 3D image of maxillary and mandibular arches from existing plaster models or impressions thus making them a practical replacement for traditional plaster models (1). 3D digital models are used for several purposes such as storage, diagnosis and the fabrication of customised appliances. 3D digital model scanning is an indirect imaging technique where the physical plaster model or impression is scanned by a laser scanner and subsequently reconstructed as a digital file. Digital model scanners occupy little space and require minimum maintenance compared to physical models. Furthermore, the scanners and their digital files provide more convenient access to plaster study models (2).

Digital models may be accurate representations of dental anatomy, but they have the limitation of showing only the crown and occlusal surfaces of the teeth and they cannot show the true size, location, or relationships of the roots of the teeth and other anatomical details unless they are correlated with conventional radiographs (e.g., panoramic, lateral or posterior-anterior cephalometric X-rays) or CBCT imaging.

By using the Ortho Insight 3D® high-resolution laser scanner on either models or impressions in conjunction with Ortho Insight 3D® Software (Motion View LLC, Chattanooga, Tennessee., USA) different analyses on dental arches can be made. These include measurements of dental arch dimensions, space analysis and Bolton tooth size discrepancies analysis. After the scanning of models or impressions, the electronically produced images do not only include the crowns of the teeth but also indicate their roots with the use of specific mathematical/geometric predictions. To the best of our knowledge, the existing bibliography does not contain any study that has evaluated the accuracy of this prediction. On the other hand, many studies have evaluated the accuracy of CBCT images with reference to different teeth locations (3-5) and reference data has been even established regarding normal crown and root lengths and root-crown ratios (6).

Hence, the assessment of the accuracy of root morphology prediction derived from digital models elaborated by the Ortho Insight 3D® software can be tested by comparison with root morphology images derived from CBCT registrations.

The aim of this study was to assess the accuracy of digital models generated by a commercially available software in predicting anterior teeth root inclination characteristics by comparison to relevant data obtained from CBCT images. The null hypothesis was that there are no differences in root inclination as predicted by commercially available 3D laser scanner software and the inclination derived from CBCT data.

## Material and Methods

-Subjects

In this retrospective study pre-treatment maxillary and mandibular plaster models and the corresponding CBCT scans of 31 consecutive patients attending a private orthodontic clinic in Beirut, Lebanon, who fulfilled the following inclusion/exclusion criteria, were selected. All subjects were medically fit and healthy, in permanent dentition, with normal tooth crown morphology. Patients who had anterior edentulous regions (more than one tooth missing), with abnormal tooth morphology, history of tooth trauma, history of previous orthodontic treatment, presence of restoration that altered the mesio-distal width of the crown, congenitally missing teeth, history of interproximal reduction and craniofacial anomalies or syndromes were excluded. The selected patients were 10 males and 21 females with age range 12 to 40 years (mean: 19.2 years; S.D.: 8.12 years).

-Materials

Each plaster model was scanned by the first author with the Ortho Insight 3D® scanner (Motion View LLC, Chattanooga, Tennessee, USA) with the resolution set at ‘‘high’’ using the supplied software (version 6.0.7044).

CBCT scans were taken using a Kodak 9500 Cone Beam 3D System (Carestream Health, Inc., Rochester, New York, USA) according to the following settings: 206 mm x 184 mm large field of view, 0.3 mm slice thickness, 60 – 90 kV tube voltage pulsed mode, 2–15 mA tube electric power, 140 kHz frequency, focal point at 0.7 mm, 2 mn and 20 sec. reconstruction time. The images obtained were converted to DICOM (Digital Imaging and Communications in Medicine) format and analyzed using the Viewbox software, version 4.1.0.1 BETA (dHAL Software, Kifissia, Greece).

CBCT scans had been obtained at the private orthodontic clinic only when there had been appropriate reasons indicating their utility over alternatives (7).

This research was approved by the Research and Ethics Committee of Hamdan Bin Mohammed College of Dental Medicine, Mohammed Bin Rashid University of Medicine and Health Sciences (Ref: EC0315-004).

-Methods

Evaluation of plaster models and CBCT data included only anterior teeth because they are simpler in terms of morphology of both their crowns and roots (6).

**Figure 1:**
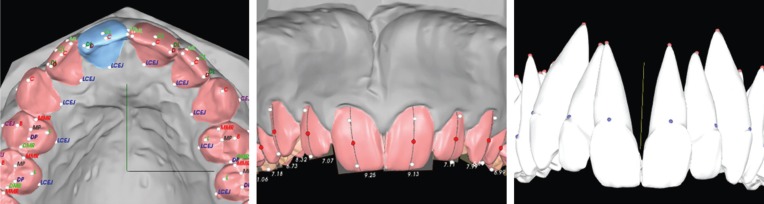
Workflow of Ortho Insight 3D® software. *Left*: landmark placement for segmenting teeth. *Center*: Setting of facial axes. *Right*: Constructed virtual roots, ready for export as STL file.

Elaboration of data took place according to the following steps:

Each patient’s maxillary and mandibular plaster models were scanned using the Ortho Insight 3D® laser scanner. Following the software’s instructions, the first author placed landmarks on the digital models (eight points for incisors and three points for canines) and identified the facial axes. The software then constructed virtual roots (Fig. 1). The tooth models, including the roots, were exported as stereo-lithography (STL) files.

The models were then loaded into the Viewbox software. The corresponding CBCT images were also loaded and a mesh representing the crowns of the teeth was created by thresholding the CBCT images. The digital models were aligned to the CBCT mesh in two stages; an initial manual alignment followed by a refined adjustment performed automatically using the Iterative Closest Point algorithm (ICP) (8).

**Figure 2:**
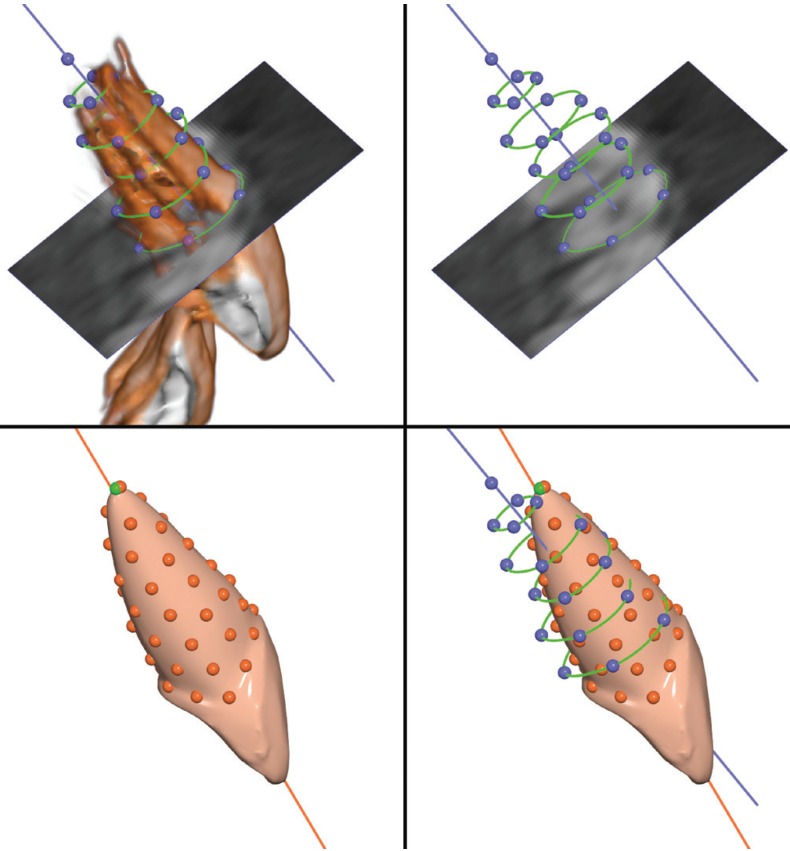
Workflow for axes angle measurement. *Upper left*: Rendering of the upper central incisor from the CBCT image. The axial slice is shown here at the level of the cervix of the tooth, but could be repositioned along the long axis. Green curves show root outlines drawn on the slice at predefined positions. Blue line is the best-fit line to the blue landmarks, representing the long axis of the root. *Upper right*: The outlines were drawn on the slices as described in the text. Each outline had four or five equidistant landmarks. *Lower left*: The incisor with virtual constructed root, as imported from the Ortho Insight 3D® software. Fifty points have been placed uniformly over the root and the best-fit line to these points represents the virtual root axis. *Lower right*: The two axes superimposed. The depicted angle is approximately 8 degrees. All images from the Viewbox software.

To determine the line representing the long axis of the root of a tooth on the digital models, the best fit line to 50 points placed uniformly on the root surface was computed (Fig. 2). The apex point was not used in the computation due to the potentially large variation in its position.

To compute the long axis of a root in the CBCT images, cross sectional slices of the root perpendicular to the long axis of the tooth were created. At predefined positions along the long axis (0%, 20%, 40%, 60%, and 80% from the cervix to the apex) the root outline was drawn and points were automatically placed on each outline. A best-fit line was computed for these points, representing the long axis of the tooth for the CBCT image (Fig. 2).

Finally, the angle between the two axes, namely the one derived from digital models and the one from the CBCT images, was computed (axes angle, AA) (Fig. 2).

-Sample size calculation

**Figure 3:**
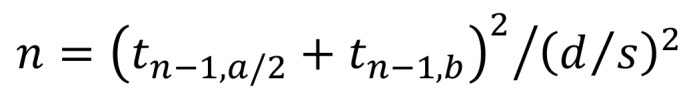
Formula.

Sample size was calculated using the Dupont and Plummer formula (9): (Fig. 3).

where s is the estimated standard deviation (here 5 degrees, based on pilot data), d is the minimum difference to be detected, set here to 3 degrees, and tα,k is the critical value that will be exceeded with probability α by a t statistic with k degrees of freedom. Using an alpha of 0.05 and a power of 80%, the formula gives a total sample of 24, which we increased to 31 for more reliable results.

-Statistical methods

Data were entered in an Excel file (Microsoft Corp. Redmond, Washington, USA), and SPSS for Windows (version 20.0, SPSS Inc., Chicago, Illinois, USA) was used for the statistical tests. Measurements of AA were tested for normality using the Shapiro-Wilk test. The Mann-Whitney and Kruskal-Wallis tests were used for comparisons between groups. A *p*-value of 0.05 or less was considered significant in all statistical analyses. In cases of multiple testing, the *p* value was adjusted using the Bonferroni correction.

-Method error

In order to evaluate intra-examiner error, records of five randomly selected patients were re- measured by the first author at an interval of two weeks. The Bland-Altman method (10) was used to compute the 95% levels of agreement (LoA); paired t-tests were used for assessing systematic error.

## Results

Paired t-tests for evaluating systematic error between the repeated measurements did not reveal any statistically significant differences. The 95% upper and lower LoA did not exceed 4.5 degrees, except for the lower left canine which was slightly larger (LoA: -4.6 to 5.8 degrees).

Axes angle (AA) was found to be not normally distributed for four out of the 12 teeth, and evidence of non-normality was present for two more. Based on these results, and considering that a non-normal distribution was to be expected, as negative values were not possible, we based our analysis on non-parametric tests.

**Figure 4:**
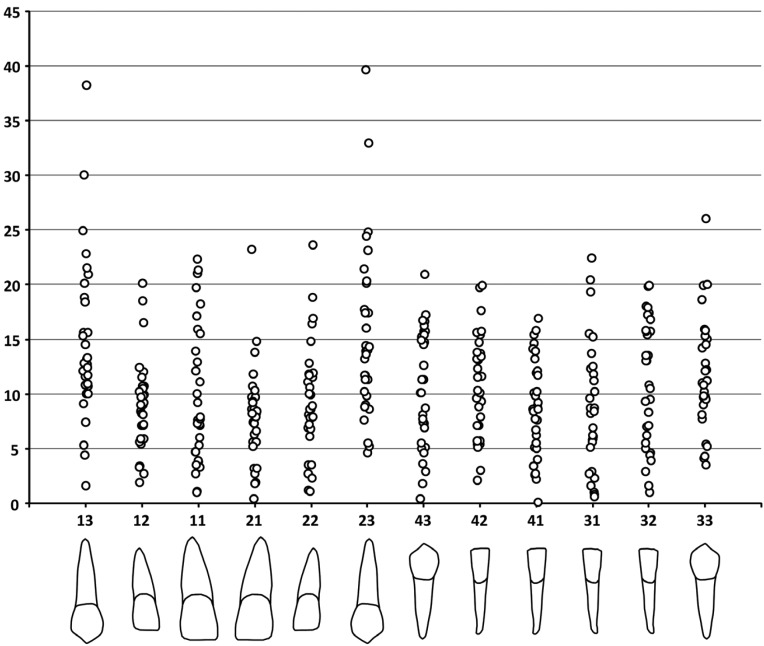
Univariate plots of the axes angles. Values are in degrees.

Descriptive statistics of the AA data are shown in  Table 1. The maximum angle between the root axes derived from digital models and CBCT data was almost 40 degrees (upper canines). The canines produced the worst results, followed by the lower lateral incisors. The upper central incisors exhibited the best results, although the maximum angle exceeded 20 degrees (but the median was only around 8 degrees) (Fig. 4).

**Table 1:**
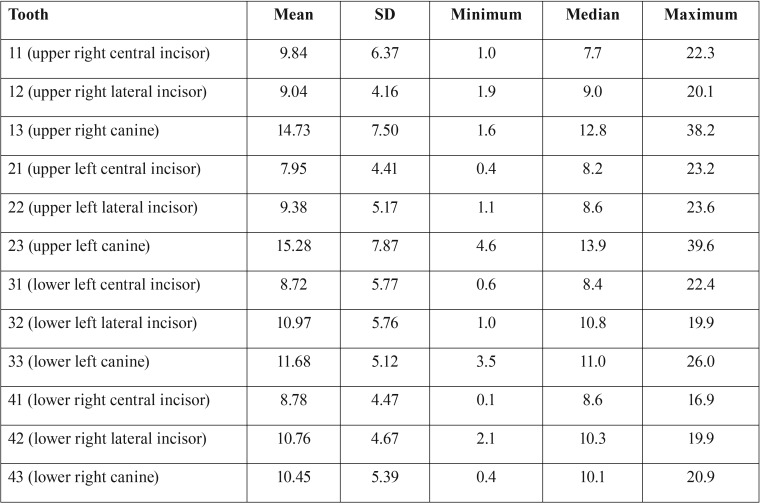
Descriptive statistics of the axes angle (AA) per tooth (N=31).

No statistically significant differences were found when comparing males to females (Mann-Whitney U test, *P* values adjusted for multiple comparisons), or between the three Angle classification groups (Kruskal Wallis test).

## Discussion

To analyze the validity of root inclination prediction of digital models derived from Ortho Insight 3D® software, CBCT was chosen as a reference standard in this study. The introduction of CBCT has considerably expanded the scope of imaging in the orthodontic clinical environment (11). CBCT provides accurate measurements, at a 1-to-1 image-to-reality scale, and avoids superimposition of structures, facilitating diagnosis of difficult clinical situations (12).

No previous research appears to have been carried out on the accuracy of root inclination prediction that uses a mathematical algorithm to construct virtual anatomical roots from digital scanning of the clinical crown. On the other hand, it has been shown that linear anatomical measurements are not significantly different in digital models when compared to CBCT images when tooth crowns and dental arches were used as reference structures for measurements (13). Since no study has compared tooth root morphology characteristics between images from digital models and CBCT, it is a reasonable aim to carry out such an investigation by considering data from the latter process. From an ideal perspective, the real roots of normal teeth constitute the “gold standard” in terms of measurement. However, there are practical and ethical factors that make forming such a control group very difficult.

Since the primary purpose of the commercially available software has been promoted and marketed as its usefulness in assessments and measurements derived from teeth crowns landmarks, the selection of the root inclination variable in this investigation was deliberately chosen as a key test of accuracy.

The variable of root inclination is simple to define, easy to understand and has meaningful orthodontic applications (e.g., evaluation of root parallelism before removal of fixed appliances).

The finding of this study showed a large range of discrepancies in the angle between the images derived from digital models and CBCT data, reaching almost 40 degrees in extreme cases (upper left canine). It is evident from the results that significant errors are to be expected. In addition, the assessment of root inclination differences between images of digital models and CBCT data means that other important morphological features such as root length, volume, shape, were not included. Furthermore, the data from the results of this investigation do not indicate the direction of the angulation error, i.e. whether it was in mesio-distal or labio-lingual direction. Errors of 10 degrees in estimating mesio-distal or labio-lingual inclination should be considered clinically significant.

Previous studies using CBCT have demonstrated relative accuracy and reliability in measurements of root length when compared to periapical radiographs (14) and to direct measurements of extracted teeth (15). However, with regard to CBCT this also depends on the voxel density range. Kim *et al.* (16) reported that CBCT measurements of root lengths may have been significantly shorter than direct measurements under a higher density range. Measurement in this investigation of only the angle between root images derived from digital models and CBCT data clearly overcomes this limitation.

The commercially available software which has been used in this investigation, has received excellent reviews regarding its use in providing dental arch measurements as well as in performing space analyses, tooth size discrepancy evaluation and other orthodontic applications based on landmarks derived from tooth crown morphology (17). Root morphology imaging prediction is not a function that this software has been designed to provide and this study verifies its limitation in routine clinical applications when used alone. However, simulation of root morphology based on scanning of plaster dental models, or dental impressions is an initial additional step, which would prove very advantageous in the future, if the accuracy of the prediction can be significantly improved. At present, these predictions cannot be considered accurate and reliable unless they are correlated with a radiographic image. If this kind of software could be given the ability to predict all the required criteria available for evaluation in a radiograph, without the need to expose the patient to radiation, it would provide significant clinical benefits.

## Conclusions

The results of the study lead to the following conclusions:

A median value for the angle between true root position derived from CBCT and estimated position ranging from 7.7 to 13.9 degrees was found, but errors of more than 20 degrees are not uncommon.

Root inclination errors were higher for upper and lower canines and lower for upper and lower central incisors.

No differences were found among different Angle classification groups.

No differences were found between the different gender and age groups.
